# Supplementary material for: Optimal Timing of External Ventricular Drainage after Severe Traumatic Brain Injury: A Systematic Review
Source: J Clin Med. 2020 Jun 25;9(6):1996. doi: 10.3390/jcm9061996 (PMC7356750; doi:10.3390/jcm9061996)
Supplement: Supplementary file 1 [file jcm-09-01996-s001.pdf]

Table S1. Search Strategy (Ovid MEDLINE(R) and Epub Ahead of Print, In-Process & Other Non-Indexed Citations, Daily and Versions(R)).

	Search Terms	Results
1	traumatic brain injury.mp.	
2	traumatic brain injury.tw.	
3	severe traumatic brain injury.tw.	
4	sTBI.mp.	
5	sTBI.tw.	
6	TBI.mp.	
7	TBI.tw.	
8	brain injury.mp.	
9	brain injury.tw.	
10	head injury.mp.	
11	head injury.tw.	
12	head trauma.mp.	
13	head trauma.tw.	
14	brain trauma.mp.	
15	brain trauma.tw.	
16	cerebral trauma.mp.	
17	cerebral trauma.tw.	
18	cerebral injury.mp.	
19	cerebral injury.tw.	
20	craniocerebral trauma.mp	
21	craniocerebral trauma.tw	
22	cranial trauma.mp	
23	cranial trauma.tw	
24	cranial injury.mp	
25	cranial injury.tw	
26	1 or 2 or 3 or 4 or 5 or 6 or 7 or 8 or 9 or 10 or 11 or 12 or 13 or 14 or 15 or 16 or 17 or 18 or 19 or 20 or 21 or 22 or 23 or 24 or 25	103098
27	intracranial hypertension.mp.	
28	intracranial hypertension.tw.	
29	refractory intracranial hypertension.mp.	
30	intracranial pressure management.mp.	
31	intracranial pressure management.tw.	
32	ICP control.mp.	
33	ICP-lowering.mp.	
34	27 or 28 or 29 or 30 or 31 or 32 or 33	10321
35	cerebrospinal fluid drainage.mp.	
36	cerebrospinal fluid drainage.tw.	
37	CSF drainage.mp.	
38	CSF drainage.tw.	
39	external ventricular drain.mp.	
40	external ventricular drain.tw.	
41	external ventricular drainage.mp.	
42	external ventricular drainage.tw.	
43	ventriculostomy.mp.	

44	ventriculostomy.tw.	
45	35 or 36 or 37 or 38 or 39 or 40 or 41 or 42 or 43 or 44	5753
46	26 and 34 and 45	107
47	limit 46 to (english language and humans)	87

Table S2. Studies with Biased Population and Reasons for Exclusion.

Author, year	Theme	Early/ Late	Reasons for bias	Study Type
**Lee, 1998** [S1]	ICP monitoring	Early	Included only patients with diffuse axonal injury (which is a subgroup of TBI patients that fitted a strict clinical and radiographic diagnosis)	Prospective observational
**Kelly, 1999** [S2]	Sedation (propofol vs morphine)	Early	Reported data for patients receiving sedation according to titration schedule; CSF drainage (step 1) precedes sedation (step 2) in the stepwise control of ICP. & Exclusion criteria of study based on medications (e.g. trial drugs not administered within/ for a certain period of time)	RCT
**Joseph, 2004** [S3]	Decompressive laparotomy	Early	Retrospective review of patients who underwent decompressive laparotomy, used when all other ICP-lowering methods have failed. These patients are expected to have worse outcomes.	Retrospective observational
**Salim, 2004** [S4]	High frequency percussive ventilation in adult respiratory distress syndrome (ARDS)	Early	Included patients only if they have sTBI, EVD, and ARDS. ARDS in sTBI may increase ICP and reduce CPP	Retrospective observational
**Olivecrona, 2007** [S5]	DC	Early	Reported data for patients receiving DC, an intervention after CSF drainage	Retrospective observational
**Oddo, 2009** [S6]	Hyperosmolar therapy	Early	Analysed patients who received osmotherapy to treat RICH (ICP >20 mmHg for >10 minutes despite initial medical management). CSF drainage is part of the initial medical management	Retrospective observational

**Rubiano, 2009** [S7]	Early DC	Early	Reported the data of 16 patients out of 179 patients who had received ventriculostomy to analyse the effectiveness of early DC	Retrospective observational
**Marshall, 2010** [S8]	Barbiturates (pentobarbital coma (PBC))	Early	Selected and analysed patients with ICP monitors AND who received PBC for treatment of RICH despite failure of best medical treatment. As all these patients are resistant to medical therapies, they may have worse outcome.	Retrospective observational
**Weiner, 2010** [S9]	DC	Early	Selected patients who had medically intractable intracranial hypertension, and underwent a delayed DC for elevated ICP	Retrospective observational
**Shi, 2015** [S10]	DC + EVD	Early	Selected patients who had severe craniocerebral trauma patients with acute post-traumatic cerebral hemispheric brain swelling (ACHS).	Retrospective observational
**Jagannatha, 2016** [S11]	Hyperosmolar therapy (mannitol vs hypertonic saline)	Early	Reported data for patients receiving osmotherapy, but osmotherapy is a second line therapy, used if ICP remained elevated >20 mmHg for > 10 minutes despite CSF drainage; Study failed to report those with CSF drainage but no osmotherapy	RCT
**Honeybyul, 2012** [S12]	DC and post-traumatic hydrocephalus	Late	Analysed patients who had a DC; DC is considered if ICP could not be maintained below 20 mm Hg despite maximal medical management	Retrospective observational
**Ho, 2014** [S13]	DC and blood brain barrier disruption	Late	Selected patients who required DC after sTBI and EVD. DC is considered if ICP could not be maintained below 20 mm Hg despite maximal medical management	Retrospective observational

CSF, cerebrospinal fluid; DC, decompressive craniectomy; EVD, external ventricular drain; ICP, intracranial pressure; RCT, randomised controlled study; RICH, refractory intracranial hypertension; (s)TBI, (severe) traumatic brain injury.

S1. Lee, T.T.; Galarza, M.; Villanueva, P.A. Diffuse axonal injury (DAI) is not associated with elevated intracranial pressure (ICP). *Acta neurochirurgica* **1998**, *140*, 41-46.

S2. Kelly, D.F.; Goodale, D.B.; Williams, J.; Herr, D.L.; Chappell, E.T.; Rosner, M.J.; Jacobson, J.; Levy, M.L.; Croce, M.A.; Maniker, A.H., et al. Propofol in the treatment of moderate and severe head injury: a randomized, prospective double-blinded pilot trial. *Journal of neurosurgery* **1999**, *90*, 1042-1052, doi:10.3171/jns.1999.90.6.1042.

S3. Joseph, D.K.; Dutton, R.P.; Aarabi, B.; Scalea, T.M. Decompressive laparotomy to treat intractable intracranial hypertension after traumatic brain injury. *The Journal of trauma* **2004**, *57*, 687-693; discussion 693-685.

S4. Salim, A.; Miller, K.; Dangleben, D.; Cipolle, M.; Pasquale, M. High-frequency percussive ventilation: an alternative mode of ventilation for head-injured patients with adult respiratory distress syndrome. *The Journal of trauma* **2004**, *57*, 542-546.

S5. Olivecrona, M.; Rodling-Wahlstrom, M.; Naredi, S.; Koskinen, L.O. Effective ICP reduction by decompressive craniectomy in patients with severe traumatic brain injury treated by an ICP-targeted therapy. *Journal of neurotrauma* **2007**, *24*, 927-935, doi:10.1089/neu.2005.356E.

S6. Oddo, M.; Levine, J.M.; Frangos, S.; Carrera, E.; Maloney-Wilensky, E.; Pascual, J.L.; Kofke, W.A.; Mayer, S.A.; LeRoux, P.D. Effect of mannitol and hypertonic saline on cerebral oxygenation in patients with severe traumatic brain injury and refractory intracranial hypertension. *Journal of neurology, neurosurgery, and psychiatry* **2009**, *80*, 916-920, doi:10.1136/jnnp.2008.156596.

S7. Rubiano, A.M.; Villarreal, W.; Hakim, E.J.; Aristizabal, J.; Hakim, F.; Diez, J.C.; Pena, G.; Puyana, J.C. Early decompressive craniectomy for neurotrauma: an institutional experience. *Ulusal travma ve acil cerrahi dergisi = Turkish journal of trauma & emergency surgery : TJTES* **2009**, *15*, 28-38.

S8. Marshall, G.T.; James, R.F.; Landman, M.P.; O'Neill, P.J.; Cotton, B.A.; Hansen, E.N.; Morris, J.A., Jr.; May, A.K. Pentobarbital coma for refractory intra-cranial hypertension after severe traumatic brain injury: mortality predictions and one-year outcomes in 55 patients. *The Journal of trauma* **2010**, *69*, 275-283, doi:10.1097/TA.0b013e3181de74c7.

S9. Weiner, G.M.; Lacey, M.R.; Mackenzie, L.; Shah, D.P.; Frangos, S.G.; Grady, M.S.; Kofke, A.; Levine, J.; Schuster, J.; Le Roux, P.D. Decompressive craniectomy for elevated intracranial pressure and its effect on the cumulative ischemic burden and therapeutic intensity levels after severe traumatic brain injury. *Neurosurgery* **2010**, *66*, 1111-1118; discussion 1118-1119, doi:10.1227/01.Neu.0000369607.71913.3e.

S10. Shi, L.; Sun, G.; Qian, C.; Pan, T.; Li, X.; Zhang, S.; Wang, Z. Technique of Stepwise Intracranial Decompression Combined with External Ventricular Drainage Catheters Improves the Prognosis of Acute Post-Traumatic Cerebral Hemispheric Brain Swelling Patients. *Frontiers in human neuroscience* **2015**, *9*, 535-535, doi:10.3389/fnhum.2015.00535.

S11. Jagannatha, A.T.; Sriganesh, K.; Devi, B.I.; Rao, G.S. An equiosmolar study on early intracranial physiology and long term outcome in severe traumatic brain injury comparing mannitol and hypertonic saline. *Journal of clinical neuroscience : official journal of the Neurosurgical Society of Australasia* **2016**, *27*, 68-73, doi:10.1016/j.jocn.2015.08.035.

S12. Honeybul, S.; Ho, K.M. Incidence and risk factors for post-traumatic hydrocephalus following decompressive craniectomy for intractable intracranial hypertension and evacuation of mass lesions. *Journal of neurotrauma* **2012**, *29*, 1872-1878, doi:10.1089/neu.2012.2356.

S13. Ho, K.M.; Honeybul, S.; Yip, C.B.; Silbert, B.I. Prognostic significance of blood-brain barrier disruption in patients with severe nonpenetrating traumatic brain injury requiring decompressive craniectomy. *Journal of neurosurgery* **2014**, *121*, 674-679, doi:10.3171/2014.6.Jns132838.

Table S3. Potentially relevant ongoing clinical trials.

#	Clinical Trials	URL
1	Assessing the Accuracy and the Impact of Standard-practice Ventricular Drainage on Intracranial Pressure Measurements Following Traumatic Brain Injury (Dual ICP)	https://clinicaltrials.gov/ct2/show/NCT02911675
2	Decompressive craniectomy for mass effect in severe head injury	http://www.isrctn.com/ISRCTN20139421
3	Bactericidal External ventricular drainS in paTients with Traumatic Brain Injury (BEST-TBI)	http://www.anzctr.org.au/ACTRN12614001175662.aspx
4	Markers of Brain injury study (MOBI)	http://www.anzctr.org.au/ACTRN12612000899842.aspx

Table S4. Risk of Bias Assessment of Included Studies.

Randomised Controlled Trials (Cochrane Collaboration's Risk of Bias Tool)		
Study	*Study/Domain*	*Risk of Bias Assessment*
Kerr, 2001 [28]	Random sequence generation	Low
	Allocation concealment	Unclear
	Blinding	Unclear
	Blinding of outcome	Unclear
	Incomplete data	Low
	Selective reporting	Low
	Other sources of bias	Low
Dizdarevic, 2012 [33]	Random sequence generation	Low
	Allocation concealment	Unclear
	Blinding	High
	Blinding of outcome	High
	Incomplete data	Low
	Selective reporting	Low
	Other sources of bias	Low
**Non-randomised studies/ Observational Studies (ROBINS-I)**		
Study	*Study/ Domain*	*Risk of Bias Assessment*
Rosner, 1995 [27]	Bias caused by confounding	Serious
	Bias of selection of participants into the study	Low
	Bias in classification of interventions	Low
	Bias caused by deviations from intended intervention	NI
	Bias caused by missing data	Moderate
	Bias in measurement of outcomes	Moderate
	Bias in selection of the reported result	Low
	Overall	Serious
Pillai, 2004 [29]	Bias caused by confounding	Serious
	Bias of selection of participants into the study	NI
	Bias in classification of interventions	Low
	Bias caused by deviations from intended intervention	NI
	Bias caused by missing data	NI
	Bias in measurement of outcomes	Serious
	Bias in selection of the reported result	Low
	Overall	Serious
Kinoshita, 2006 [30]	Bias caused by confounding	Serious
	Bias of selection of participants into the study	NI
	Bias in classification of interventions	Serious
	Bias caused by deviations from intended intervention	NI
	Bias caused by missing data	NI
	Bias in measurement of outcomes	Serious
	Bias in selection of the reported result	Low
	Overall	Serious
Timofeev, 2008 [1]	Bias caused by confounding	Moderate
	Bias of selection of participants into the study	Low
	Bias in classification of interventions	Serious

	Bias caused by deviations from intended	NI
	intervention	Low
	Bias caused by missing data	Low
	Bias in measurement of outcomes	Moderate
	Bias in selection of the reported result	Serious
	Overall	
	Bias caused by confounding	Moderate
	Bias of selection of participants into the study	Low
	Bias in classification of interventions	Critical
	Bias caused by deviations from intended	NI
Griesdale, 2010 [31]	intervention	Low
	Bias caused by missing data	Serious
	Bias in measurement of outcomes	Low
	Bias in selection of the reported result	Critical
	Overall	
	Bias caused by confounding	Serious
	Bias of selection of participants into the study	Low
	Bias in classification of interventions	Serious
	Bias caused by deviations from intended	NI
Zeng, 2010 [32]	intervention	Low
	Bias caused by missing data	Serious
	Bias in measurement of outcomes	Moderate
	Bias in selection of the reported result	Serious
	Overall	
	Bias caused by confounding	Serious
	Bias of selection of participants into the study	Low
	Bias in classification of interventions	Low
	Bias caused by deviations from intended	NI
de Andrade, 2011 [34]	intervention	Low
	Bias caused by missing data	Serious
	Bias in measurement of outcomes	Low
	Bias in selection of the reported result	Serious
	Overall	
	Bias caused by confounding	Serious
	Bias of selection of participants into the study	Serious
	Bias in classification of interventions	Moderate
	Bias caused by deviations from intended	NI
Kasotakis, 2012 [35]	intervention	Serious
	Bias caused by missing data	Moderate
	Bias in measurement of outcomes	Moderate
	Bias in selection of the reported result	Serious
	Overall	
	Bias caused by confounding	Moderate
	Bias of selection of participants into the study	Low
	Bias in classification of interventions	Moderate
	Bias caused by deviations from intended	NI
Yuan, 2013 [36]	intervention	Low
	Bias caused by missing data	Serious
	Bias in measurement of outcomes	Low
	Bias in selection of the reported result	Serious
	Overall	
Nwachuku, 2014 [37]	Bias caused by confounding	Moderate

	Bias of selection of participants into the study	NI
	Bias in classification of interventions	Low
	Bias caused by deviations from intended	NI
	intervention	NI
	Bias caused by missing data	Critical
	Bias in measurement of outcomes	Moderate
	Bias in selection of the reported result	Critical
	Overall	
	Bias caused by confounding	Serious
	Bias of selection of participants into the study	Low
	Bias in classification of interventions	Moderate
	Bias caused by deviations from intended	NI
Childs, 2015 [38]	intervention	Low
	Bias caused by missing data	Serious
	Bias in measurement of outcomes	Moderate
	Bias in selection of the reported result	Serious
	Overall	
	Bias caused by confounding	Low
	Bias of selection of participants into the study	Low
	Bias in classification of interventions	Low
	Bias caused by deviations from intended	NI
Liu, 2015 [39]	intervention	Moderate
	Bias caused by missing data	Serious
	Bias in measurement of outcomes	Low
	Bias in selection of the reported result	Serious
	Overall	
	Bias caused by confounding	Low
	Bias of selection of participants into the study	Low
	Bias in classification of interventions	Moderate
	Bias caused by deviations from intended	NI
Khalili, 2016 [40]	intervention	Serious
	Bias caused by missing data	Serious
	Bias in measurement of outcomes	Low
	Bias in selection of the reported result	Serious
	Overall	
	Bias caused by confounding	Serious
	Bias of selection of participants into the study	Low
	Bias in classification of interventions	Moderate
	Bias caused by deviations from intended	NI
Akbik, 2017 [41]	intervention	Serious
	Bias caused by missing data	Moderate
	Bias in measurement of outcomes	Low
	Bias in selection of the reported result	Serious
	Overall	
	Bias caused by confounding	Moderate
	Bias of selection of participants into the study	Low
	Bias in classification of interventions	Serious
Aiolfi, 2018	Bias caused by deviations from intended	Low
	intervention	Low
	Bias caused by missing data	Low
	Bias in measurement of outcomes	Moderate
	Bias in selection of the reported result	Serious

	Overall	
Klein, 2018 [42]	Bias caused by confounding	Moderate
	Bias of selection of participants into the study	NI
	Bias in classification of interventions	Low
	Bias caused by deviations from intended intervention	NI
	Bias caused by missing data	Moderate
	Bias in measurement of outcomes	Moderate
	Bias in selection of the reported result	Low
	Overall	Moderate
Bales, 2019	Bias caused by confounding	Low
	Bias of selection of participants into the study	Low
	Bias in classification of interventions	Moderate
	Bias caused by deviations from intended intervention	Low
	Bias caused by missing data	Low
	Bias in measurement of outcomes	Low
	Bias in selection of the reported result	Moderate
	Overall	Moderate
Lescot, 2012 [2]	Bias caused by confounding	Serious
	Bias of selection of participants into the study	Serious
	Bias in classification of interventions	Low
	Bias caused by deviations from intended intervention	NI
	Bias caused by missing data	Serious
	Bias in measurement of outcomes	Serious
	Bias in selection of the reported result	Low
	Overall	Serious
Bhargava, 2013 [18]	Bias caused by confounding	Serious
	Bias of selection of participants into the study	Low
	Bias in classification of interventions	Critical
	Bias caused by deviations from intended intervention	NI
	Bias caused by missing data	Low
	Bias in measurement of outcomes	Low
	Bias in selection of the reported result	Low
	Overall	Critical
